# Evolution of the *Neopsylla hongyangensis* Mitogenome: Insights Into the Mitogenomic Evolution of the Orders Siphonaptera and the Phthiraptera

**DOI:** 10.1002/ece3.71108

**Published:** 2025-03-11

**Authors:** Xiaoxia Lin, Ju Pu, Wenge Dong

**Affiliations:** ^1^ Yunnan Provincial Key Laboratory for Zoonosis Control and Prevention, Institute of Pathogens and Vectors Dali University Dali Yunnan China

**Keywords:** evolution, mitogenome, *Neopsylla hongyangensis*, phylogeny, plague

## Abstract

The evidence that parasitic animals exhibit elevated mitogenomic evolution rates is inconsistent and limited to Arthropoda. *Neopsylla hongyangensis* Li, Bai et Chen, 1986 (Siphonaptera: Ctenophthalmidae) feeds on the host's blood and is an important medical insect with plague transmission. In this study, we sequenced the *N. hongyangensis* mitogenome and explored the mitogenomic evolution of Siphonaptera and Phthiraptera, which both belong to the Insecta on warm‐blooded animals. The mitogenomes of Siphonaptera are closed‐circular double‐stranded DNA molecules and exhibit highly conserved structural features. In contrast, the mitogenomes of most Phthiraptera species exhibit extensive fragmentation and comprise multiple minichromosomes. We performed a comparative analysis of nucleotide composition, Ka/Ks ratios, and codon usage patterns in Siphonaptera and Phthiraptera mitogenomes. Compared to Phthiraptera with low locomotory capacity, Siphonaptera with high locomotory capacity have higher AT content, slower evolution, and greater influence from natural selection (i.e., micro‐habitat). The mitogenomic evolution of Siphonaptera and Phthiraptera was influenced by locomotory capacity and life history. Phylogenetic analysis based on 13 PCGs showed that Ceratophyllidae, Leptopsyllidae, and Ctenophthalmidae were paraphyletic, and Vermipsyllidae, Hystrichopsyllidae, Pulicidae, and Pygiopsyllidae were monophyletic. This study provides new insights into the phylogenetic relationships and mitogenomic evolution of Siphonaptera.

## Graphical abstract

The *Neopsylla hongyangensis* is an important medical insect that transmits plague. We sequenced the mitogenome of *N*. *hongyangensis* and constructed a phylogenetic tree for the order Siphonaptera. In addition, we explored the mitogenomic evolution of the orders Siphonaptera and Phthiraptera, which both belong to the class Insects, on warm‐blooded animals based on base composition, Ka/Ks, and codon preference.

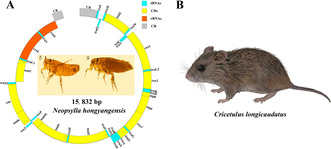

## 1 Introduction

Plague, a lethal zoonotic disease that is primarily transmitted by fleas (Perry and Fetherston 1997). Approximately 2574 flea species belonging to 16 families and 238 genera were described worldwide (Bitam et al. 2010), among which 257 flea species are associated with plague (Medvedev et al. 2019). *Neopsylla hongyangensis* Li, Bai et Chen, 1986 belongs to the class Insecta, order Siphonaptera, family Ctenophthalmidae, and genus *Neopsylla*. The *N. hongyangensis* has polyxenous species, primarily on *Cricetulus* followed by *Rattus* and *Apodemus* (Li et al. 1995). The *N. hongyangensis* can transmit plague ( *Yersinia pestis* ) and is an important medical insect (Dubyanskiy and Yeszhanov 2016). The genus *Neopsylla* was divided into four species groups based on the number of pronotum bristles: *setosa*‐group, *bidentatiformis*‐group, *stevensi*‐group, and *anoma*‐group (Liu 1986). The *N. hongyangensis* was placed within the *setosa*‐group according to the bottle‐shaped arm protruding backward on the ventral surface of sternum IX (Li et al. 1995). However, some researchers believed that the bottle‐shaped arm structure may be determined by the genotype and thus suggested that *N. hongyangensis* may be a synonym of *N. bidentatiformis*, placing it within the *bidentatiformis*‐group (Lu and Wu 2001). It can be seen that the two species groups are very similar, and later Wu (2007) recognized them as a single species group, that is, *setosa*‐group (Wu 2007). Mitogenome is the most frequently and widely used molecular marker to assess evolutionary relationships between species (Chen et al. 2012). To date, only 23 species of flea mitogenomes are available, and the *N. hongyangensis* mitogenome is still a gap. Obtaining mitogenomes of various flea species will enhance our understanding of the evolutionary relationship within Siphonaptera.

At present, the five dominant ectoparasites of warm‐blooded animals are fleas, lice, ticks, chigger mites, and gamasid mites, respectively. Among these, only fleas and lice belong to the Insecta. Fleas are usually acquired from nests or infected individuals, while lice are acquired through interindividual transmission (Benedek et al. 2024). Fleas and lice share certain adaptations in terms of haematophagy, which may lead to aggregation distribution, and lice demonstrate a higher degree of aggregation than fleas (Zuo and Guo 2011). Fleas are capable of transmitting a range of diseases, including plague ( *Y. pestis* ), murine typhus (*Rickettsia mooseri*) and cat‐scratch disease ( *Bartonella henselae* ) (Bitam et al. 2010). Lice are capable of transmitting a range of diseases, including relapsing fever ( *B. quintana* ), epidemic typhus ( *R. prowazekii* ) and plague ( *Y. pestis* ) (Bland et al. 2024; Brouqui and Raoult 2006). Ectoparasites exhibit variability in host specificity, microhabitat preference, and transmission patterns (Hopla et al. 1994; Paramasvaran et al. 2009). The life history of fleas includes four stages: eggs, larvae, pupae, and adults, which belong to holometabolous insects, and only adult fleas migrate onto hosts (Bitam et al. 2010). The life history of lice includes three stages: eggs, nymphal, and adult, which belong to incomplete holometabolous insects, and all stages are on hosts (Kim et al. 1986). Periodic ectoparasite fleas are more sensitive to microhabitat than permanent ectoparasite lice (Little et al. 2024). A previous study showed significant variation in mitogenome structures between the two groups. The Siphonaptera mitogenomes are closed‐circular double‐stranded DNA molecules and exhibit highly conserved structural features, whereas the mitogenomes of most Phthiraptera exhibit extensive fragmentation and comprise multiple minichromosomes (Cameron et al. 2011).

In this study, (i) we sequenced the mitogenome of *N. hongyangensis* with plague transmission for the first time; (ii) we constructed a phylogenetic tree among various species in Siphonaptera and evaluated the evolutionary relationships among different lineages; (iii) we compared structural features and variations of the mitogenomes between Siphonaptera and Phthiraptera and analyzed the evolutionary rates of their mitogenomes; (iv) we investigated the influence of locomotory capacity and life history on the mitogenomic evolution of both Siphonaptera and Phthiraptera.

## 2 Materials and Methods

### 2.1 Sample Collection and Morphological Identification

**FIGURE 1:**
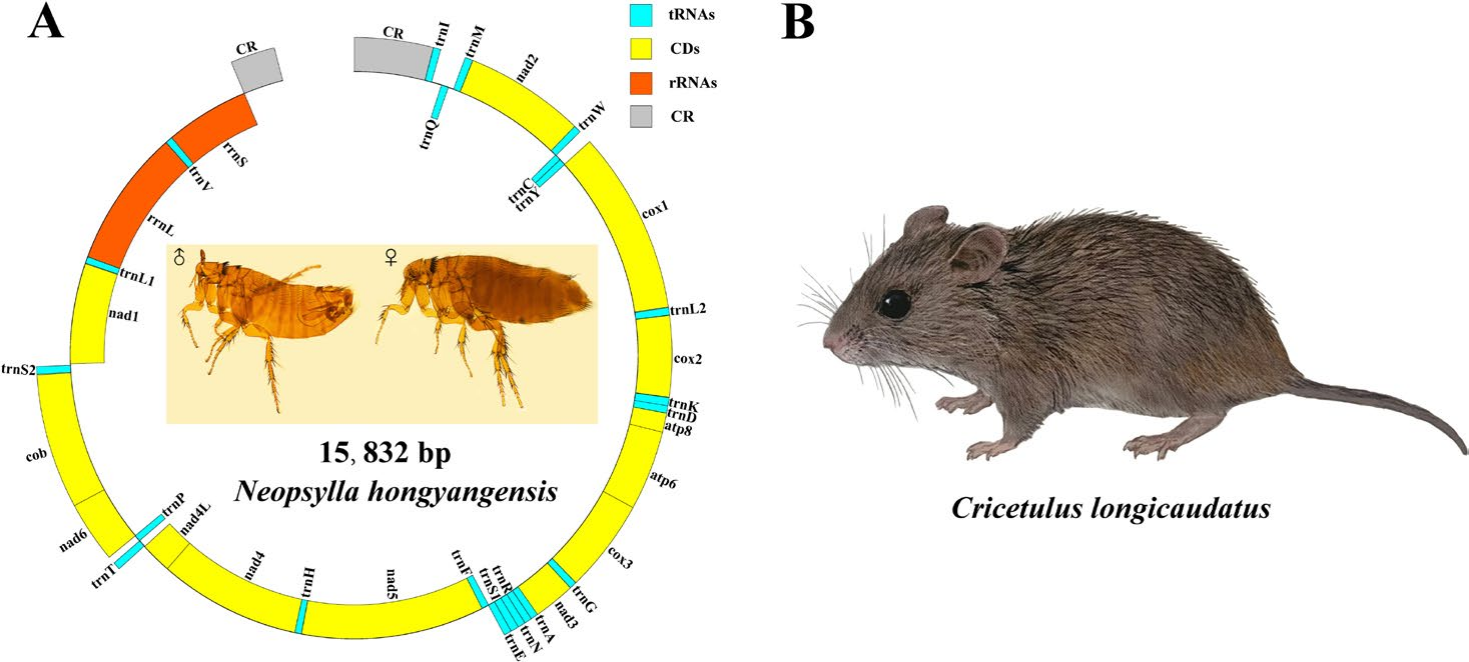
(A) Circular map of mitogenome of *Neopsylla hongyangensis* (external genes are encoded by the heavy (H) strand, in contrast to the internal genes that are encoded by the light (L) strand); (B) Host of *Neopsylla hongyangensis*.

Adult specimens of *N. hongyangensis* were collected from *Cricetulus longicaudatus* in Hongyuan County, Aba Tibetan and Qiang Autonomous Prefecture, Sichuan Province, China (32°48′37′′N, 102°32′27′′E) (Figure 1B). All specimens were stored in 95% ethanol at −80°C until further processing. Morphological identification of the species was performed based on the identifying features described in the *Fauna Sinica Insecta Siphonaptera* (Wu 2007). Morphological features of *N. hongyangensis* were photographed using an SZ2‐ILST dissecting microscope (Olympus, Tokyo, Japan). Specimens were deposited at the Institute of Pathogen and Vector Biology, Dali University. This study was approved by the Animal Ethics Committee of Dali University (approval no. MECDU‐201912‐20).

### 2.2 Mitogenome Sequencing, Assembly and Annotation

The specimens were sent to Shanghai Winnerbio Technology Co. Ltd. (Shanghai, China) for DNA extraction and sequencing. After the purity and integrity of the amplified DNA were tested, a DNA library with an insert fragmented by sonication size of 450 bp was constructed, followed by paired‐end sequencing on an Illumina NovaSeq 6000 platform (Modi et al. 2021). By fastp (https://github.com/OpenGene/fastp) software for low‐quality data filtering, 5.44G raw data after filtering obtained 5.29 G clean data (GC content is 35.1%, Q20 value is 98.59%, Q30 value is 95.9%). The clean data were then de novo assembled using MitoZ v. 2.3 (https://doi.org/10.1101/489955), resulting in a graph file, which was visualized using the Bandage software (Wick et al. 2015) to assemble the *N. hongyangensis* mitogenome. To ensure assembly quality, the raw data was mapped to the assembled genome using BWA v.0.7.17 (https://bio‐bwa.sourceforge.net/) and Samtools v.0.1.20 (https://github.com/samtools/samtools/releases?page=2), and the total sequencing depth was evaluated. Sequencing depth should generally be ≥ 100×, which means that the assembly results have high accuracy. The sequencing depth was 978.515X, which guaranteed high single‐base correctness of the *N. hongyangensis* mitogenome.

The mitogenome sequence obtained by splicing was uploaded to the MITOS webserver (Bernt et al. 2013) for preliminary annotation to determine the approximate location of each gene. Protein‐coding genes (PCGs) were identified by open reading frame (ORF) searches in Geneious Prime v.11.1.5 (Kearse et al. 2012) using the invertebrate mt genetic code and alignment with published flea species of the same genus (Liu et al. 2023). tRNAscan‐SE v.2.0 (Chan et al. 2021) and ARWEN v.1.2.3 (Laslett and Canbäck 2008) were used to identify and infer the secondary structures of 22 tRNA genes in *N. hongyangensis*. Mitogenome circular visualization was performed with the web server GenomeVx (http://wolfe.ucd.ie/GenomeVx/).

### 2.3 Sequence Analysis

All publicly available mitogenome sequences of Siphonaptera and Phthiraptera were obtained from NCBI (https://www.ncbi.nlm.nih.gov). The selected complete mitogenome sequences (24 Siphonaptera and 28 Phthiraptera) used for analysis (Table 1). The nucleotide composition of the mitogenome was calculated using MEGA v.11.0 (Tamura et al. 2021). Skewness values were calculated according to the formulas: AT‐skew = [A − T]/[A + T] and GC‐skew = [G − C]/[G + C]. DnaSP v.6.0 (Librado and Rozas 2009) was used to analyze the non‐synonymous substitution rate (Ka) and synonymous substitution rate (Ks) for 13 PCGs in each species.

**TABLE 1:** Mitogenome sequences of the order Siphonaptera and Phthiraptera analysis in the present study.

Order	Suborder	Family	Subfamily	Species	GenBank accession number
Siphonaptera		Pulicidae	Pulicinae	*Ctenocephalides felis*	MT594468
				*Ctenocephalides felis felis*	MW420044
				*Ctenocephalides canis*	MW234554
				*Ctenocephalides orientis*	NC073009
				*Pulex irritans*	MW745784
				*Xenopsylla cheopis*	MW310242
		Hystrichopsyllidae	Hystrichopsyllinae	*Hystrichopsylla weida qinlingensis*	NC042380
		Ctenophthalmidae	Rhadinopsyllinae	*Stenischia humilis*	NC073020
				*Stenischia montanis yunlongensis*	OR780663
			Ctenophthalminae	*Ctenophthalmus quadratus*	NC072692
				*Ctenophthalmus yunnanus*	OR780664
			Neopsyllinae	*Neopsylla specialis*	NC073019
				*Neopsylla hongyangensis*	*PP133648*
			Stenoponiinae	*Stenoponia polyspina*	OR834393
		Pygiopsyllidae	Stivallinae	*Aviostivalius klossi bispiniformi*	OR774970
		Vermipsyllidae		*Dorcadia ioffi*	NC036066
		Ceratophyllidae	Ceratophyllinae	*Ceratophyllus wui*	NC040301
				*Ceratophyllus anisus*	NC073017
				*Jellisonia amadoi*	NC022710
				*Macrostylophora euteles*	OR774969
		Leptopsyllidae	Leptopsyllinae	*Frontopsylla diqingensis*	OR780662
				*Frontopsylla spadix*	NC073018
				*Leptopsylla segnis*	NC072691
				*Paradoxopsyllus custodis*	OQ627398
Phthiraptera	Anoplura	Haematopinidae		*Haematopinus apri*	KC814611‐19
				*Haematopinus asini*	KJ434034‐38; KF939318; KF939322; KF939324; KF939326
				*Haematopinus suis*	KC814602‐10
				*Haematopinus tuberculatus*	ON416547‐16556
		Pedicinidae		*Pedicinus badii*	MT721726–39
				*Pedicinus obtusus*	MT792495–506
				*Pediculus schaeffi*	KC241882‐97; KR706168‐69
				*Pediculus humanus capitis*	JX080388‐407
		Polyplacidae		*Polyplax asiatica*	KF647751‐61
				*Polyplax spinulosa*	KF647762‐72
				*Polyplax reclinata*	MW291451‐61
		Hoplopleuridae		*Hoplopleura sp*	MT792483‐94
		Linognathidae		*Linognathus vituli*	OL677823‐32
		Microthoraciidae		*Microthoradus praelongiceps*	KX090378‐89
	Ischnocera	Philopteridae		*Columbicola passerinae*	MT094266‐82
				*Bothriometopus macrocnemis*	EU183542
				*Campanulotes compar*	MH001225
				*Coloceras sp*	JN122000
				*Ibidoecus bisignatus*	NC015999
				*Falcolipeurus quadripustulatus*	NC039529
				*Falcolipeurus suturalis*	MW696813
		Trichodectidae		*Trichodectes canis*	MH001213‐24; MH823541
	Amblycera	Menoponidae		*Amyrsidea minuta*	MH001227
				*Colpocephalum griffoneae*	NC039530
				*Menacanthus cornutus*	OM718871
				*Osborniella crotophagae*	MW199175
		Boopidae		*Heterodoxus macropus*	AF270939
				*Heterodoxus spiniger*	MW199168

CodonW v.1.4.2 (https://sourceforge.net/projects/codonw/) was used to calculate codon bias parameters, including RSCU, ENC, A3s, U3s, C3s, G3s, and GC3s. GC12 values were calculated using a Python script. Relative synonymous codon usage (RSCU) was mainly used to measure the relative probability of specific codons encoding corresponding amino acids among synonymous codons, which could intuitively reflect codon preference (Yuan et al. 2023). The ENC‐GC3s plot assesses the factors affecting codon usage bias in different species by using the Effective number of codons (ENC) as the ordinate and GC3s as the abscissa. The standard curve reflects the theoretical ENC values that are only caused by mutations, which is based on the equation: ENC_expected_ = 2 + GC3s + (29/GC3s^2^ + [1 + GC3s])^2^ (Wright 1990). The neutral plot assesses the extent to which random mutation and selection pressure influence codon usage by plotting GC12 as the ordinate and GC3s as the abscissa. If the dots are distributed along the diagonal with a regression coefficient approaching 1, it indicates that mutations play a more important role in codon usage bias. When dots are scattered and the regression coefficient approaches 0, it suggests that natural selection is the more important factor (Sueoka 1988). PR2 analysis was used to calculate A3s/(A3s + U3s) and G3s/(G3s + C3s) by A3s, U3s, G3s, and C3s, respectively. The central position of the scatter plot (A = T, C = G) represents codon usage without bias. Correspondence analysis (COA) was used to analyze the differences in codon usage bias among different species and to further explore differences in species evolution and pressures. The ENC‐GC3s plot, neutral plot, and COA analysis were visualized using the ggplot2 and ggpmisc packages in R Studio v.4.1.0. The PR2 plot was visualized using ggplot2, Biostrings, and seqinr packages in R Studio v.4.1.0 (Team R 2019).

### 2.4 Phylogenetic Analysis

Phylogenetic trees of Siphonaptera were constructed for 13 PCGs of the mitogenome sequences of 24 fleas, covering 7 families (Table 1), with *Boreus elegans* of Mecoptera as the outgroup. All steps of phylogenetic analysis were performed on Phylosuite v.1.2.3 software (Zhang et al. 2020). The PCGs were aligned using G‐INS‐i algorithms implemented in MAFFT v.7.0 (Katoh and Standley 2013). Ambiguously aligned positions were trimmed using Gblocks v.0.91 (Castresana 2000). ModelFinder (Kalyaanamoorthy et al. 2017) was used to select the best‐fit partition model based on the Bayesian information criterion (BIC). Maximum Likelihood (ML) and Bayesian analyses (BI) were inferred using IQ–TREE v.1.6.1 (Nguyen et al. 2015) and MrBayes v.3.2.7a (Ronquist et al. 2012), respectively. The ML tree was selected by 5000 ultrafast bootstrap replicates. For the BI tree, four independent Markov chains (MCMC) were run for 1,000,000 generations and sampled every 1000 generations, with the first 25% discarded as burn‐in. Convergence was considered to be reached when the estimated sample size (ESS) exceeded 100 and the potential scale reduction factor (PSRF) approached 1.0. The trees were visualized and summarized in FigTree v.1.4.4 (http://tree.bio.ed.ac.uk/software/figtree/).

## 3 Results

### 3.1 Mitogenome Structure

In this study, the nearly complete mitogenome, with the exception of the partial non‐coding region of *N. hongyangensis*, was sequenced, annotated, and submitted to NCBI (GenBank accession no. PP133648) for the first time. The nearly complete mitogenome of *N. hongyangensis* was 15,832 bp in size, and the base composition was A = 37.2%, T = 37.4%, C = 16.0%, G = 9.4%, a significant AT bias (74.6%) and a negative AT‐skew (−0.002) and GC‐skew (−0.258) (Table 2). The *N. hongyangensis* mitogenome is a circular double‐stranded DNA molecule, which contains a control region (CR) and 37 conserved genes (13 PCGs, 22 tRNAs and 2 rRNAs). The mitogenome sequence is identical to that of *Drosophila yakuba* (Clary and Wolstenholme 1985), and there is no rearrangement (Figure 1A).

**TABLE 2:** Nucleotide composition and skewness of the *Neopsylla hongyangensis* mitogenome.

Gene	A%	T%	C%	G%	AT%	AT‐skew	GC‐skew
*nad1*	29.4	46	7.4	17.2	75.4	−0.22	0.397
*nad2*	33.5	44.4	14	8	77.9	−0.14	−0.274
*nad3*	27.1	43	20.8	9.1	70.1	−0.228	−0.39
*nad4*	29.8	44.7	9.3	16.2	74.5	−0.2	0.269
*nad4L*	32.7	46.9	5.1	15.3	79.6	−0.179	0.5
*nad5*	30.9	43.4	8.9	16.8	74.3	−0.169	0.311
*nad6*	36	43.2	13.8	7	79.2	−0.09	−0.327
*cox1*	27.3	37.3	19.7	15.6	64.6	−0.154	−0.116
*cox2*	32.5	36.7	19.5	11.3	69.2	−0.062	−0.267
*cox3*	28.5	38.4	19.3	13.8	66.9	−0.149	−0.166
*atp6*	31.5	39.9	17.9	10.7	71.4	−0.117	−0.25
*atp8*	38.1	42.9	13.7	5.4	81	−0.059	−0.438
*cob*	29.5	39.9	19	11.6	69.4	−0.15	−0.243
PCGs	30.5	41.8	14.3	13.5	72.3	−0.156	−0.03
rRNAs	39.2	40.3	6.5	13.9	79.5	−0.013	0.363
tRNAs	40.1	38.1	10.1	11.8	78.2	0.026	0.075
Whole genome	37.2	37.4	16	9.4	74.6	−0.002	−0.258

### 3.2 Protein‐Coding Genes and RNA Genes

The majority of 13 PCGs in *N. hongyangensis* are located on the heavy (H) strand, with only four genes (*nad1*, *nad4*, *nad4L*, *nad5*) located on the light (L) strand (Figure 1A, Table 3). The total length of 13 PCGs was 11,144 bp, accounting for 70.39% of the mitogenome. All PCGs of *N. hongyangensis* used ATN as the start codon, and all PCGs used TAA or TAG as stop codons except for *nad5* and *nad4* with incomplete T—as stop codons (Table 3). These incomplete stop codons are adjacent to tRNAs at the 3′ ends, which are believed to be formed by post‐transcriptional polyadenylation of tRNA (Yokobori and Pääbo 1997).

**TABLE 3:** Organization of the *Neopsylla hongyangensis* mitogenome.

Gene	Strand	Location	Size	Anticodon	Start/stop codon	Intergenic nucleotides
*trnI*	H	662–724	63	GAU		
*trnQ*	L	820–888	69	UUG		95
*trnM*	H	938–1003	66	CAU		49
*nad2*	H	1004–2014	1011		ATC/TAA	
*trnW*	H	2013–2076	64	UCA		−2
*trnC*	L	2069–2129	61	GCA		−8
*trnY*	L	2130–2192	63	GUA		
*cox1*	H	2190–3725	1536		ATC/TAA	−3
*trnL2*	H	3730–3793	64	UAA		4
*cox2*	H	3795–4475	681		ATG/TAA	1
*trnK*	H	4478–4547	70	CUU		2
*trnD*	H	4547–4608	62	GUC		−1
*atp8*	H	4609–4776	168		ATC/TAA	
*atp6*	H	4770–5441	672		ATG/TAA	−7
*cox3*	H	5441–6223	783		ATG/TAA	−1
*trnG*	H	6224–6285	62	UCC		
*nad3*	H	6286–6636	351		ATC/TAG	
*trnA*	H	6635–6697	63	UGC		−2
*trnR*	H	6698–6760	63	UCG		
*trnN*	H	6761–6824	64	GUU		
*trnS1*	H	6825–6893	69	UCU		
*trnE*	H	6894–6957	64	UUC		
*trnF*	L	6956–7023	68	GAA		−2
*nad5*	L	7024–8740	1717		ATG/T	
*trnH*	L	8742–8803	62	GUG		1
*nad4*	L	8804–10,142	1339		ATG/T	
*nad4L*	L	10,136–10,429	294		ATG/TAA	−7
*trnT*	H	10,436–10,500	65	UGU		6
*trnP*	L	10,501–10,563	63	UGG		
*nad6*	H	10,562–11,077	516		ATT/TAA	−2
*cob*	H	11,077–12,210	1134		ATG/TAA	−1
*trnS2*	H	12,216–12,280	65	UGA		5
*nad1*	L	12,295–13,236	942		ATG/TAG	14
*trnL1*	L	13,242–13,303	62	UAG		5
*rrnL*	L	13,304–14,599	1296			
*trnV*	L	14,600–14,666	67	UAC		
*rrnS*	L	14,666–15,456	791			−1
incomplete CR		1–661; 15,457–15,832	661; 376			

**FIGURE 2:**
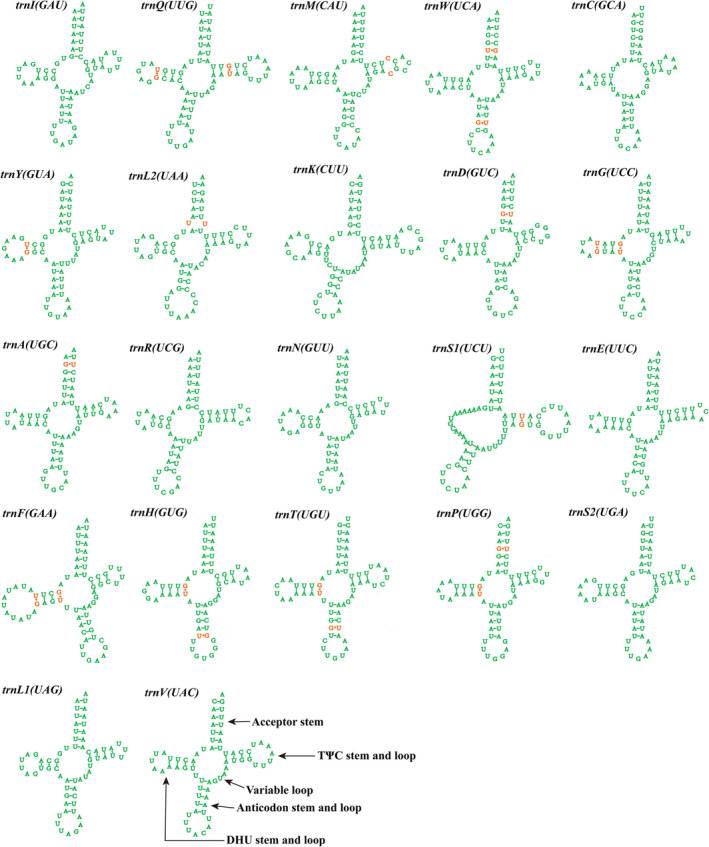
Secondary structure of 22 tRNA genes from the *Neopsylla hongyangensis*.

The length of the tRNA gene in the *N. hongyangensis* mitogenome ranges from 61 to 70 bp, with the exception of *trnS*_*1*_(tct), which lacks a DHU arm; the remaining 21 tRNAs all have typical cloverleaf structures. There are 20 mismatches among the 22 tRNAs, including 18 G–U weak mismatches and 1 each of C–C and U–U strong mismatches (Figure 2). The *rrnL* and *rrnS* genes in *N. hongyangensis* are 1296 and 791 bp in size, respectively. The *rrnL* is located between *trnL*_*1*_(tag) and *trnV*, and *rrnS* is located between *trnV* and CR (Figure 1A).

### 3.3 Base Composition

**FIGURE 3:**
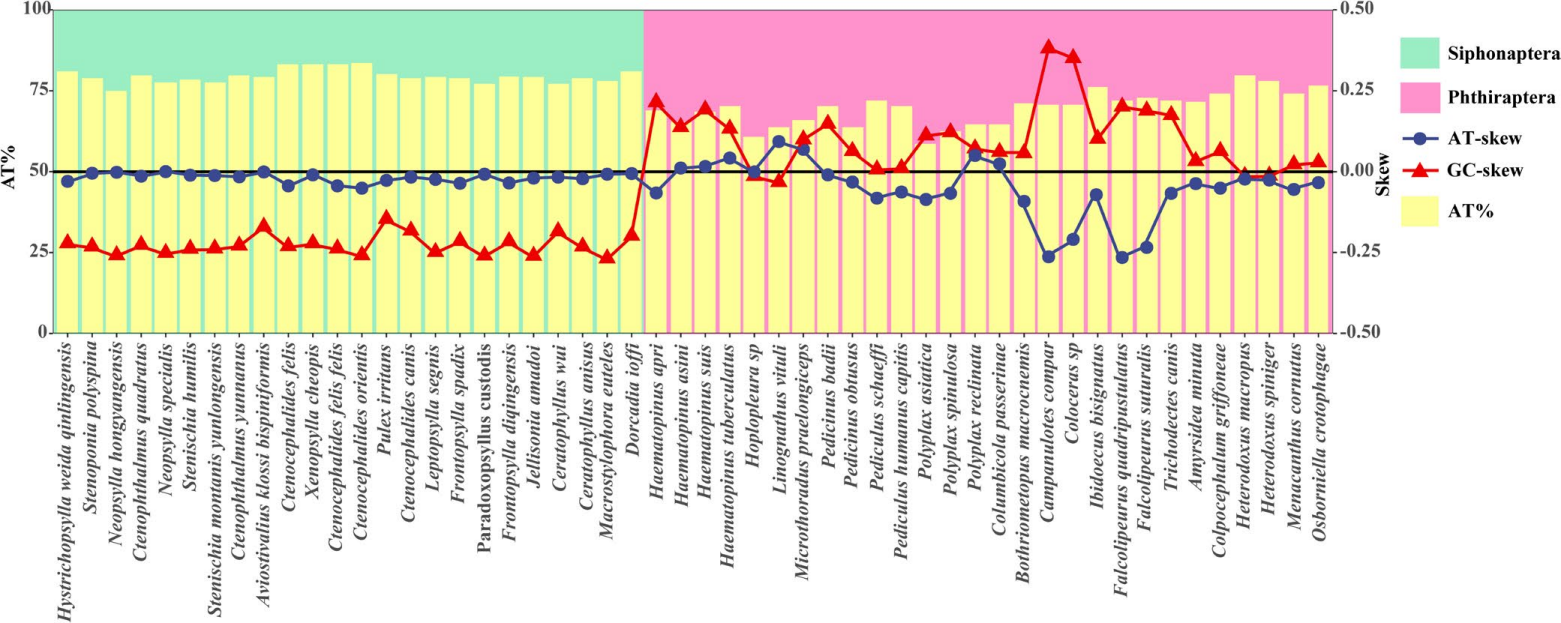
Base composition analysis of the order Siphonaptera and Phthiraptera.

The AT content, AT‐skew, and GC‐skew were calculated and analyzed for 52 species (24 order Siphonaptera and 28 order Phthiraptera). Results showed that AT content in Siphonaptera ranged from 74.6% to 83.2%, whereas in Phthiraptera it ranged from 60.4% to 79.3%. The AT‐skew and GC‐skew values in Siphonaptera were negative, with AT‐skew ranging from −0.051 to 0, and GC‐skew ranging from −0.268 to −0.146. The overall skewness magnitude for AT‐skew was 0.051, and for GC‐skew it was 0.122 (Figure 3). In Phthiraptera, most AT‐skew values were negative (except for *Haematopinus asini* , *H. suis* , *H. tuberculatus* , *Linognathus vituli* , *Microthoradus praelongiceps*, *Polyplax reclinate*, *Columbicola passerinae*), and most GC‐skew values were positive (except for *Hoplopleura sp*, *L. vituli* , *Heterodoxus macropus*, *H. spiniger* ), with AT‐skew ranging from −0.013 to 0.381 and GC‐skew ranging from −0.268 to 0.093. The overall skewness magnitude for AT‐skew was 0.368, and for GC‐skew it was 0.175 (Figure 3). The overall skewness magnitude of both AT‐skew and GC‐skew in Siphonaptera was lower than that in Phthiraptera. Additionally, GC‐skew values in Siphonaptera were lower than those in Phthiraptera (Figure 3).

### 3.4 Comparison of Evolutionary Rates (Ka/Ks)

**FIGURE 4:**
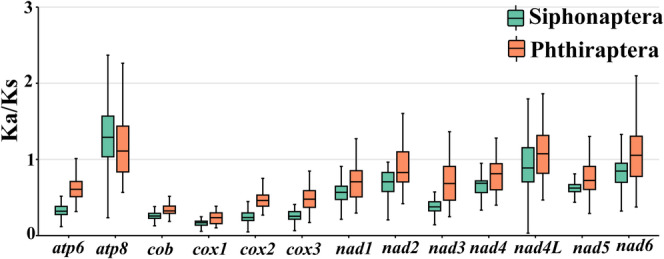
Selective pressure analysis in the protein coding genes of the order Siphonaptera and Phthiraptera.

Ka/Ks ratio of non‐synonymous (Ka) to synonymous (Ks) nucleotide substitution is used as an indicator of selective pressure on protein‐coding sequences between different sequences (Wang et al. 2009). Ka/Ks > 1 indicates positive selection, Ka/Ks = 1 indicates neutral selection, and Ka/Ks < 1 indicates purifying selection (Yang and Bielawski 2000). We calculated the average Ka/Ks ratio for each PCG across 52 species (24 Siphonaptera and 28 Phthiraptera). In Siphonaptera, the Ka/Ks ratio of each PCG was as follows: *atp8* > *nad4L* > *nad6* > *nad2* > *nad4* > *nad5* > *nad1* > *nad3* > *atp6* > *cob* > *cox3* > *cox2* > *cox1*. In Phthiraptera, the Ka/Ks ratio of each PCG was as follows: *atp8* > *nad4L* > *nad6* > *nad2* > *nad4* > *nad5* > *nad3* > *nad1* > *atp6* > *cox2* > *cox3* > *cob* > *cox1*. Both showed that *atp8* evolved the fastest, and *cox1* evolved the slowest (Figure 4). However, in Siphonaptera, only the *atp8* gene was under positive selection (Ka/Ks > 1), while the other genes were under purifying selection (Ka/Ks < 1). In Phthiraptera, three genes (*atp8*, *nad4L*, *nad6*) were under positive selection (Ka/Ks > 1), indicating that the overall evolutionary rate of Phthiraptera is faster than that of Siphonaptera (Figure 4).

### 3.5 Analysis of Codon Bias

**FIGURE 5:**
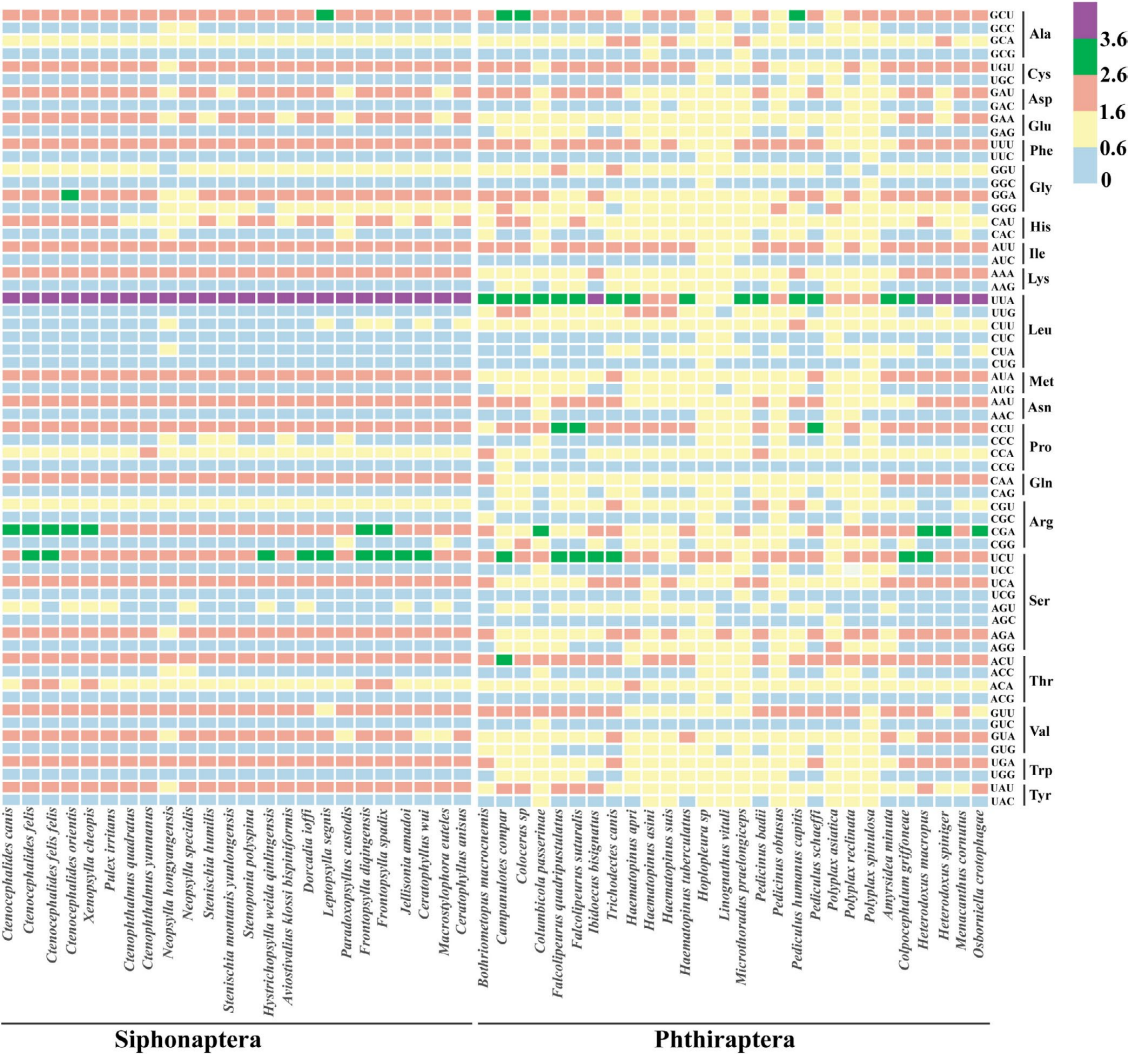
Codon usage of the order Siphonaptera and Phthiraptera mitogenomes.

RSCU analysis was performed on 13 PCGs of 52 species (24 order Siphonaptera and 28 order Phthiraptera). RSCU > 1.6 are overrepresented codons, while RSCU < 0.6 are underrepresented codons (Dharmashekara et al. 2021). In Siphonaptera, there is little variation in codon usage among different species, with an average of 22 codons being overrepresented codons, mainly composed of A or U, with UUA being the most frequently used (RSCU values all > 3.6). In Phthiraptera, there is a significant variation in codon usage among different species. For example, in *Heterodoxus macropus*, there are 23 overrepresented codons, while in *Hoplopleura sp*., there is only 1 overrepresented codon. The overrepresented codons are also mainly composed of A or U (Figure 5).

**FIGURE 6:**
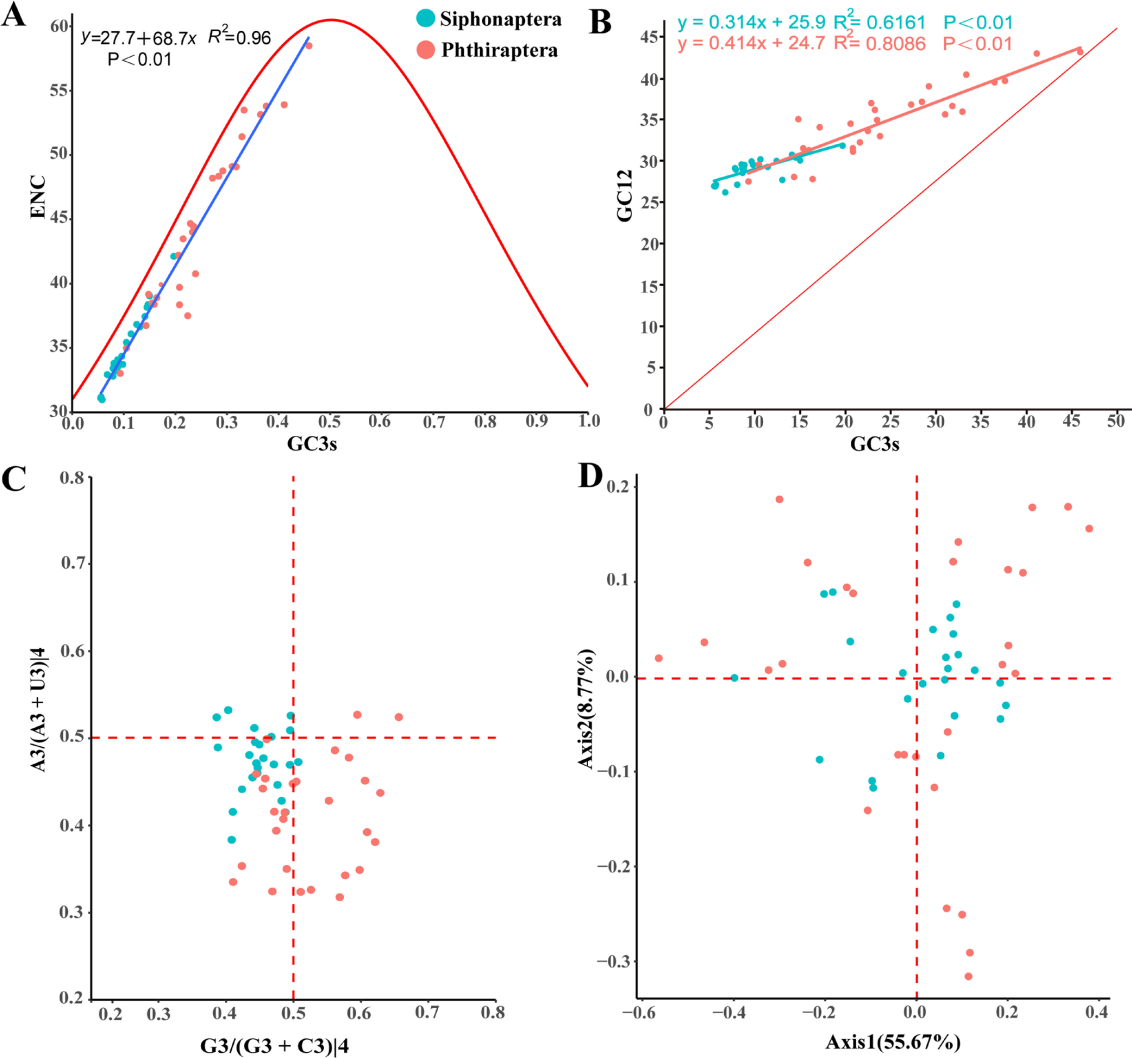
Codon usage preference analysis of 52 species in the order Siphonaptera and Phthiraptera. (A) ENC‐GC3s plot, (B) Neutral plot, (C) PR2 plot, (D) COA analysis.

ENC values range is generally 20 to 61, with lower ENC values indicating stronger codon usage bias. When ENC ≤ 35, there is significant codon bias (Lal et al. 2016). ENC values for 13 PCGs in Siphonaptera range from 30.97 to 42.12, with an average of 34.85, indicating a lower ENC value and thus stronger codon bias. ENC values for 13 PCGs in Phthiraptera range from 33.02 to 58.49, with an average of 44.38, indicating a higher ENC value and weaker codon bias. The codons for most species of Siphonaptera and Phthiraptera fall below the standard curve (Figure 6A), suggesting that natural selection is a major factor influencing codon bias.

To assess whether 13 PCGs in Siphonaptera and Phthiraptera are neutrally evolving, GC content of the first and second codon positions (GC12) and the third codon position (GC3s) were calculated. In the neutral plot, we found a positive correlation between GC3s and GC12s in both Siphonaptera and Phthiraptera (Figure 6B). The slopes of the regression line of species from Siphonaptera and Phthiraptera indicated that both mutation pressure (31.4% for Siphonaptera and 41.4% for Phthiraptera) and natural selection (68.6% for Siphonaptera and 58.6% for Phthiraptera) influenced codon bias (Figure 6B). The influence of natural selection on Siphonaptera was higher than that on Phthiraptera.

We divided these points into four quadrants centered around 0.5 for comparison and found that Siphonaptera were predominantly located in the third quadrant (A3/(A3 + U3) 4 < 0.50 and G3/(G3 + C3) 4 < 0.50), with many more points than in the other quadrants, while the first quadrant contained the fewest points. This indicates that the third codon position in Siphonaptera mitogenome follows the pattern U>A, C>G (Figure 6C). Most Phthiraptera were evenly distributed between the third and fourth quadrants, which only suggests that the third position follows the pattern U>A but did not reveal a bias for G or C (Figure 6C). The balance between A/U and G/C has been disrupted in both Siphonaptera and Phthiraptera, indicating that the formation of codon bias in their mitogenomes is influenced not only by mutation pressure but also by natural selection.

Corresponding analysis (COA) to explore the differences in codon usage among 13 PCGs across various species within Siphonaptera and Phthiraptera. Grouped comparisons showed that most Siphonaptera species clustered nearly at the origin of the two axes, with few outliers, indicating conservation. However, Phthiraptera is nearly all distant from the origin of the two axes, with many outliers, indicating greater difference in codon usage (Figure 6D).

The pattern of codon usage was often influenced by various factors during its formation, with the major factors including mutation pressure and natural selection (Chen et al. 2004; Qiu et al. 2011). Comprehensive analysis combining RSCU, ENC‐GC3s plot, neutral plot, PR2‐plot, and COA indicates that the codon usage patterns in Siphonaptera and Phthiraptera mitogenomes may be influenced by multiple factors, including mutation pressure and natural selection. Natural selection plays a dominant role in Siphonaptera and Phthiraptera, but the influence of natural selection on Siphonaptera is more pronounced than that on Phthiraptera. Additionally, the codon usage among different species of Siphonaptera exhibits minimal variation, whereas significant variation is observed among different species of Phthiraptera. These observations suggest that the Siphonaptera mitogenome is more conservative, while the Phthiraptera mitogenome is more variable.

### 3.6 Phylogenetic Analysis

**FIGURE 7:**
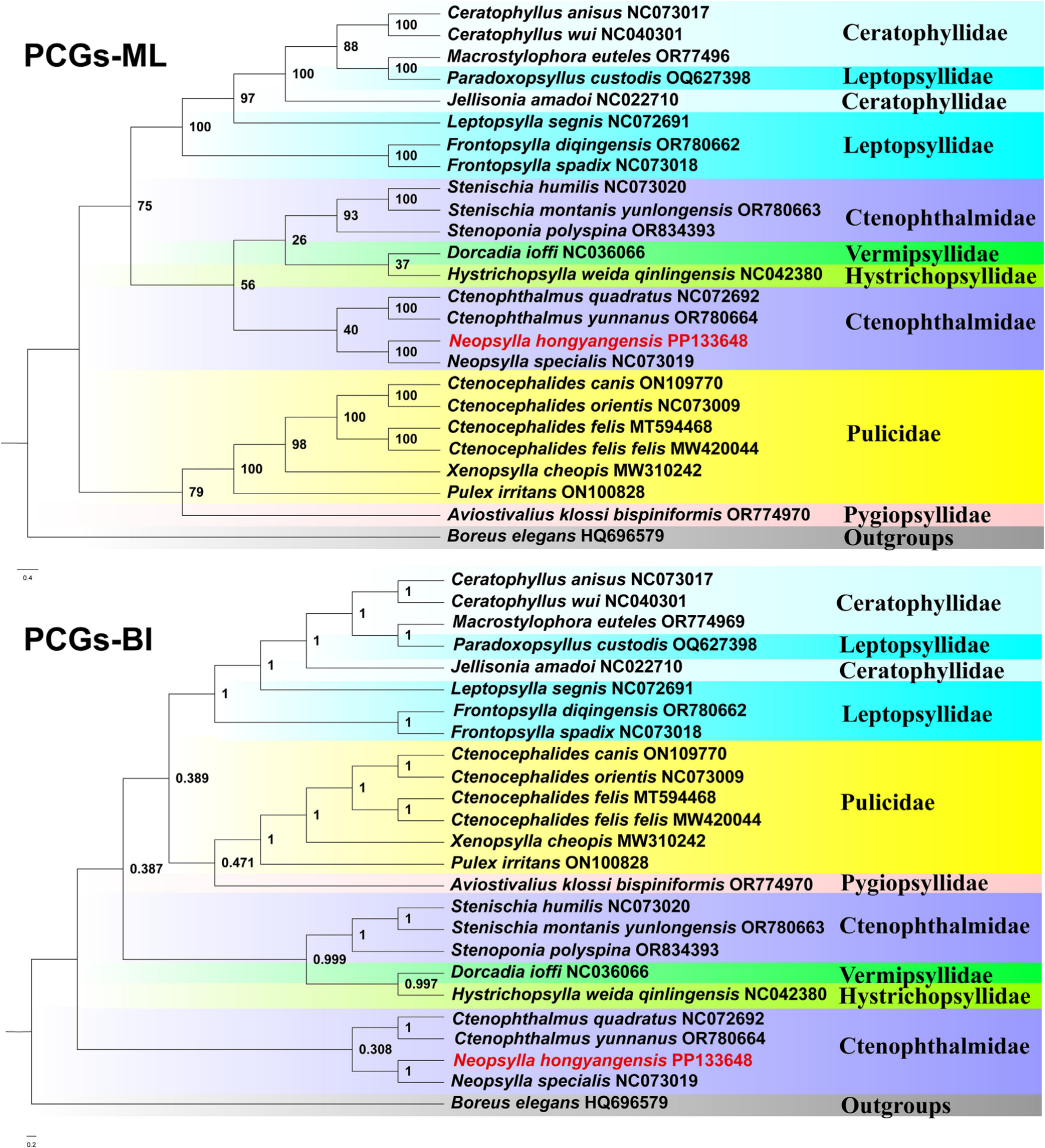
Phylogenetic relationships among 24 species of the order Siphonaptera inferred from Bayesian inference (BI) and maximum likelihood (ML) analysis of 13 PCGs.

Phylogenetic trees were constructed using the BI and ML methods for 13 PCGs from the existing Siphonaptera mitogenomes, resulting in different tree topologies with the two methods (Figure 7). Our results indicated that the families Ceratophyllidae, Leptopsyllidae, and Ctenophthalmidae are paraphyletic, while the families Vermipsyllidae, Hystrichopsyllidae, Pulicidae, and Pygiopsyllidae are monophyletic. The (*N. hongyangensis* + *N. specialis* ) from the subfamily Neopsyllinae and the (*Ctenophthalmus yunnanus* + *C. quadratus* ) from Ctenophthalminae are sister groups, but with low support (UFBoot = 29, Bpp = 0.308). The most obvious difference between the ML and BI trees is the phylogenetic position of Ctenophthalmidae:in the ML tree, Ctenophthalmidae is a sister group to ((Vermipsyllidae + Hystrichopsyllidae) + Ctenophthalmidae), while in the BI tree, it is a sister group to (((Ceratophyllidae + Leptopsyllidae) + (Pulicidae + Pygiopsyllidae)) + (Ctenophthalmidae + (Vermipsyllidae + Hystrichopsyllidae))) (Figure 7).

## 4 Discussion

As a vector for the plague, the *N. hongyangensis* mitogenome has significant AT bias (AT = 74.6%) and a highly conservative gene arrangement, consistent with the ancestral arrangement of arthropods (Clary and Wolstenholme 1985). Previous studies suggested that ‘parasitism’ would lead to species evolution quickly (Oliveira et al. 2008), but in reality, other factors can also cause species to evolve more quickly. There is an association between mitogenome evolution and locomotory capacity, which has been confirmed in fish (Strohm et al. 2015; Sun et al. 2011), insects (Mitterboeck and Adamowicz 2013), mollusks (Sun et al. 2018), birds, and mammals (Shen et al. 2009). At present, the number of available flea mitogenomes is limited since fleas with tiny and high locomotory capacity are very difficult to collect. To date, only 24 mitogenomes of fleas have been sequenced (including the *N. hongyangensis* in this study). In addition, only fleas and lice in Insecta are hematophagous ectoparasites, which can co‐evolve with their hosts, and their living environment will be more consistent. Fleas are periodic ectoparasites with high locomotory capacity, whereas lice are permanent ectoparasites with low locomotory capacity. From these differences in life history and locomotory capacity, we can explore their evolutionary divergence. We selected Siphonaptera and Phthiraptera, which both belong to Insecta on warm‐blooded animals, for comparative analysis of nucleotide composition, Ka/Ks ratios, and codon usage patterns in the Siphonaptera and Phthiraptera mitogenomes. The results showed that the AT content of Siphonaptera with high locomotory capacity was higher than that of Phthiraptera with lower locomotory capacity, and the overall skew magnitude was smaller than that of Phthiraptera. Locomotory capacity is positively correlated with energy expenditure (Jakovlić et al. 2023), while skew magnitude is negatively correlated with locomotory capacity (Jakovlić et al. 2021). This result is consistent with our findings; the higher the locomotory capacity, the more energy expenditure (AT content), and the lower the skew magnitude. Furthermore, our findings indicate that the Siphonaptera with high locomotory capacity experience stronger purifying selection, and the evolutionary rate is slower than that of Phthiraptera with lower locomotory capacity, which is consistent with the previously reported (Jakovlić et al. 2023).

Codon bias analysis was helpful for studying the evolution and environmental adaptability of various species (Behura and Severson 2013). The results of RSCU and COA analysis showed that the differences in codon usage among different species of Siphonaptera are minimal, whereas the differences among species of Phthiraptera are pronounced. This suggested that Phthiraptera has undergone a complex evolutionary radiation and has been more greatly influenced by its hosts during evolution. Furthermore, the results of the ENC‐GC3s plot, neutral plot, and PR2 plot showed that both Siphonaptera and Phthiraptera have been influenced by both natural selection and mutational pressure. Among them, natural selection played a key role in both orders, but its influence was more pronounced in Siphonaptera than in Phthiraptera. This may be attributed to the life history of Siphonaptera. The life cycles of fleas consist of four stages: egg, larva, pupa, and adult. Especially, the three stages of eggs, larvae, and pupae are easily influenced by environmental factors, and thus have a greater influence from natural selection. It supported that periodic ectoparasite fleas are more sensitive to environmental factors than permanent ectoparasite lice (Little et al. 2024).

Some researchers have proposed that rapid evolution can lead to the instability and extinction of species, while slower evolution can bring higher biodiversity (Herrera‐Flores et al. 2022). Flea species are rich, and even subspecies have appeared, which is closely related to their slower evolution. As a pest that everyone dislikes, fleas can transmit serious diseases such as plague, and their slower evolution is not a good thing. This reminds us to strengthen our research on fleas and do a good job in their control and prevention.

Based on the inferred topology from existing flea mitogenome data, the families Ceratophyllidae, Leptopsyllidae, and Ctenophthalmidae are paraphyletic, while the families Vermipsyllidae, Pulicidae, and Pygiopsyllidae are monophyletic. These findings are consistent with the results of analyses using single‐gene data (Whiting et al. 2008). In previous studies (Whiting et al. 2008), Hystrichopsyllidae was always paraphyletic. However, in the present study, due to the fact that there is only one species in Hystrichopsyllidae, it appears monophyletic. In phylogenetic trees, Ceratophyllidae is always located at the top of the phylogenetic tree, representing that Ceratophyllidae is a younger family, which is consistent with previous research findings (Zhu et al. 2015). The current results are insufficient to resolve the taxonomic status of Ctenophthalmidae for two reasons: (i) Compared to studies based on single‐gene data, the representative data used in this study are severely insufficient. The Ctenophthalmidae has nine subfamilies, but only four subfamilies have representative data. These include two species each from the subfamilies Ctenophthalminae, Neopsyllinae, and Ischnopsyllinae, and one species from the Stenopsyllinae; (ii) The phylogenetic tree for Ctenophthalmidae were unstable, as the ML and BI trees cannot produce a consistent topology, with most results having lower support. Therefore, increasing the sample size in the future is necessary to obtain reliable phylogenetic trees.

## 5 Conclusion

In this study, the *N. hongyangensis* mitogenome was reported for the first time. Comparative analysis of mtDNA evolution between Siphonaptera and Phthiraptera showed that Siphonaptera with high locomotory capacity has higher AT content, slower evolution, and greater influence from natural selection than mutation pressure, while Phthiraptera with lower locomotory capacity were opposed. The Siphonaptera and Phthiraptera mtDNA evolution is influenced mainly by locomotory capacity and life history. In addition, we reconstructed phylogenetic trees of Siphonaptera and indicated that Ceratophyllidae, Leptopsyllidae, and Ctenophthalmidae were paraphyletic, and Vermipsyllidae, Hystrichopsyllidae, Pulicidae, and Pygiopsyllidae were monophyletic, and the taxonomic status of Ctenophthalmidae was unstable. Mitogenome data provide valuable insights into the genetic mechanism of parasite–host evolutionary patterns. More mitogenome data and new methods are needed to further explore the evolutionary process in Siphonaptera.

## Author Contributions

**Xiaoxia Lin:** conceptualization (equal), data curation (equal), formal analysis (equal), methodology (equal), visualization (equal), writing – original draft (equal), writing – review and editing (equal). **Ju Pu:** investigation (equal), software (equal). **Wenge Dong:** conceptualization (equal), funding acquisition (equal), resources (equal), supervision (equal), writing – review and editing (equal).

## Conflicts of Interest

The authors declare no conflicts of interest.

## Data Availability

The mitogenome sequence data of *Neopsylla hongyangensis* were deposited in GenBank (https://www.ncbi.nlm.nih.gov/) accession no. PP133648.
